# Calcium Soaps as a Factor in Appendicitis

**Published:** 1908-10-24

**Authors:** 


### PATHOLOGY.

### CALCIUM SOAPS AS A FACTOR IN APPENDICITIS.

The prevalence of appendicitis has led to all kinds of surmises as to its causation. It is difficult to be absolutely certain that the disease is really more common than it used to be, for cases are now recorded as appendicitis which formerly would have passed away undiagnosed; but the general opinion is that there are really more cases of it than there formerly were, and it is quite certain that it is very much commoner in some countries than it is in others. Common in the United Kingdom and in the United States, for example, it is comparatively rare in Spain and Italy. There must be some reason for this, and the profession cannot but be grateful for the attempt that has been made by Dr. Owen T. Williams * to throw light upon the subject by his investigations upon fat assimilation and upon the behaviour of the calcium soaps in the intestinal canal. Dr. Williams employs the same micro-chemical tests as those used by Klotz and Lorrain Smith; the methods depend upon the following chemical facts:—

*Osmic acid* stains neutral fats black; it does not blacken palmitic and stearic acids.

*Sudan III.* and *Scharlach R.* stain neutral fats a bright pink, whereas they stain soaps a pinkish yellow. The difference in these two colours is easily seen in the specimens obtained.

*Nile-blue sulphate* stains neutral fats pink, and stains fatty acids violet.

*Silver nitrate* forms an impregnation of silver oxide on calcium compounds.

*Petroleum ether* dissolves neutral fats from a specimen, but no soaps. This can be used as a further test for differentiating these two compounds.

Microscopical sections of normal and of abnormal vermiform appendices having been treated by the above various stains, it was possible to draw conclusions as to the nature of the fats, soaps, and calcium salts in the normal organ, in the inflamed organ, in concretions within the appendix, and so on. The normal appendix used as the basis of comparison was one found accidentally in a hernial sac and thence removed. Sections of this showed a little fatty degeneration of the mucous membrane, traces of soaps in the submucosa, and some calcium deposit in the mucous membrane at certain points. "Sections from diseased appendices treated by the same methods and under the same conditions show most striking differences—differences which are so great as to be immediately obvious in sections stained in bulk and observed macroscopically. The changes cannot be due to involution changes, as they are seen in specimens obtained from cases occurring in children. These sections show a *large increase in the production of calcium soaps*, both in the mucous membrane and in the submucosa. In some of the specimens almost a complete ring of soaps is to be found in the submucosa." Thickening in the latter was found sometimes to be due mainly to the laying down of these soaps. Small quantities of fatty acids are also found in the submucosa of the diseased appendices by the Nile-blue sulphate method. Staining by silver nitrate shows the presence of calcium in definite quantity in the submucosa.

These observations show, therefore, that in appendicitis there is a marked change in the wall of the tube, a change characterised by the increased production of calcium soaps in the mucous membrane, and more particularly their formation in the submucosa. In some specimens the calcium soaps can be seen passing into the lumen of the appendix, looking like mucus, but really having a very similar composition to the concretion which results.

The question which at once arises is whether this excess of calcium soaps is caused by the inflammation, or whether it causes the inflammation, or whether both have a common cause, or whether the co-existence of excess of calcium soaps is merely accidental. The latter possibility seems to be at once put out of court by the constancy with which the submucosal and mucosal deposits of calcium soaps can be demonstrated; of the remaining three, the one favoured by Dr. Williams is the second—namely, that the deposit of calcium soaps precedes the inflammation, and indirectly causes it. This is the point to which investigators will direct further attention; though meanwhile the contention seems probable on many grounds. It is possible, for example, and, indeed, not so very uncommon, to find typical appendicular concretions at post-mortem examinations upon persons who have never had appendicular symptoms, and whose vermiform appendices look perfectly healthy and free from adhesions, as though they had never been inflamed. Such cases would be very easily explainable upon Dr. Williams' hypothesis, upon which much calcium soap might exist without appendicitis, but not *vice versâ*. That the appendicular concretions arise *in situ*, and not by intrusion of material from the bowel, is rendered extremely probable by the fact that they are concentrically striated around a central nucleus, and by the fact that the same chemical substances are present both in the concretion and in the appendicular submucous and mucous coats. The bowel can produce similar deposits within itself, and there is a close chemical similarity between the intestinal sand of mucous colitis and the concretion within a vermiform appendix: in each there is a tendency for part of the calcium soaps to go a stage further and become calcium carbonate.

The conclusions drawn by Dr. Williams are:—That from clinical evidence and pathological and chemical observations it would appear that there is produced in the course of the changes in the intestinal wall an abnormal condition associated with formation of calcium soaps. These calcium soaps when formed in the intestine in excess produce intestinal sand; associated with this there are symptoms of colic with, under certain circumstances, mucous colitis.

* *Brit. Med. Journ.*, July 27, 1907; *Bio-Chemical Journal*, vol. ii., No. 9, 1907; *Liverpool Medico-Chirurgical Journal*, vol. 54, 1908, p. 425.

This change taking place under similar conditions and probably from the same causes in the appendix, where there are poorer opportunities for the discharge of the deleterious bodies, is probably an important factor in the ætiology of appendicitis. The barrier of calcium soaps deposited in the submucosa, cutting off the nutritive supply to the already altered mucous membrane, invasion of the latter by micro-organisms becomes much easier than before.

